# Visual and instrumental coverage error of two dental shade guides: an in vivo study

**DOI:** 10.1007/s00784-022-04556-0

**Published:** 2022-05-31

**Authors:** Javier Ruiz-López, Maria M. Perez, Cristina Lucena, Rosa Pulgar, Ana López-Toruño, Maria Tejada-Casado, Razvan Ghinea

**Affiliations:** 1 Department of Optics, Faculty of Science, University of Granada, Campus de Fuente Nueva, Edificio Mecenas, s/n 18071, ibs-Granada, Granada, Spain; 2 Department of Stomatology, Faculty of Dentistry, University of Granada, Campus de Cartuja s/n, 18071 Granada, Spain; 3 Department of Physics, Faculty of Sciences, University of Craiova, 13 AI Cuza Street, 200585 Craiova, Romania

**Keywords:** Coverage error, Shade guides, Color differences, Shade matching

## Abstract

**Objectives:**

This study aims to evaluate in vivo the color agreement between natural teeth and dental shade guides by means of visual and instrumental coverage error ($\mathrm{CE}$) index.

**Materials and methods:**

The color of the middle third of 735 incisors was visually determined by two evaluators using the Vita Classical (VC) and Vita 3D Master (V3DM) shade guides. The color match between the natural tooth and the shade tab was rated as poor (P), good (G), or optimum (O) by each observer. CIE color coordinates of the target teeth and shade tabs of VC and V3DM were instrumentally measured using a clinical spectrophotometer. Visual (${\mathrm{CE}}_{\mathrm{V}}$) and instrumental (${\mathrm{CE}}_{\mathrm{I}}$) coverage error indexes were computed using CIELAB and CIEDE2000 metrics for both shade guides. For ${\mathrm{CE}}_{\mathrm{V}}$ calculation, only the concordant inter-observer determination on tooth shade rated as O–O or O–G was used. The results were evaluated using perceptibility (PT, ${\mathrm{\Delta E}}_{\mathrm{ab}}^{*}$= 1.2, ${\mathrm{\Delta E}}_{00}$= 0.8) and acceptability (AT, ${\mathrm{\Delta E}}_{\mathrm{ab}}^{*}$= 2.7, ${\mathrm{\Delta E}}_{00}$= 1.8) color thresholds for dentistry.

**Results:**

VC and V3DM exhibited ${\mathrm{CE}}_{\mathrm{I}}$ (2.5, 3.2, and 3.2, 2.7 CIELAB units; 1.9, 2.3, and 2.8, 2.4 CIEDE2000 units, respectively, for O–O and O–G match) and ${\mathrm{CE}}_{\mathrm{V}}$ (4.7, 4.8, and 4.1, 4.6 CIELAB units; 3.3, 3.4, and 3.4, 3.6 CIEDE2000 units, respectively, for O–O and O–G match) values greater than 50:50% AT for both color difference formulas. ${\mathrm{CE}}_{\mathrm{I}}$ contributes more than 50% (53.2–82.4% range) to the ${\mathrm{CE}}_{\mathrm{V}}$ value. This contribution depends on the shade guide used and the quality of the visual rating.

**Conclusions:**

The evaluated shade guides exhibited visual coverage errors above acceptability thresholds, largely due to the contribution of the instrumental coverage error to the visual coverage error.

**Clinical relevance:**

It necessary to further improve commercially available dental shade guides to facilitate achievement of satisfactory esthetics results in clinical practice.

## Introduction

Tooth color determination is a key point for the success of highly esthetic restorations and clinically is done by visual determination as well as instrumental measurements. Visual color determination is performed comparing the target tooth with the tabs of commercial shade guides. Nevertheless, this procedure is unreliable, mostly due to its high subjectivity and the ability of multiple factors to influence its performance, such as the experience of the clinician, illumination, or angle of observation [1–3]. In spite of its numerous limitations, visual shade matching still remains the most widely used in clinical practice.

On the other hand, clinical color measurement devices (such as colorimeters, spectrometers, spectrophotometers, and digital image analysis instruments) are free of observer subjectivity, thus improving the reproducibility of the shade matching procedure [4–7]. Dental color-measuring instruments provide colorimetric tooth data or report the “color” as the nominal reference shade of one or several commercial clinical shade guides [8]. However, several studies have pointed out some important limitations and inconsistencies of these tooth color-measuring devices [9–11].

Previous studies have shown that available clinical shade guides do not cover the whole color range of natural teeth [12–14]. This limitation, coupled with the remarkable capacity of the human eye to detect small color differences [15, 16], allows for easy recognition of color adjustment errors in clinical restorations, which leads to esthetic failures [2] and consequent loss of clinical productivity and efficiency.

In dentistry, the coverage error index ($\mathrm{CE}$) describes the mean of minimal color differences (${\Delta \mathrm{E}}_{\mathrm{min}}$) between the specimens of one set (e.g., a shade guide) and each specimen of another set (e.g., natural teeth) [17]. The smaller the coverage error value of a shade guide, the higher the chances of a successful shade matching with that specific guide. Despite its great practicality as a tool for assessing the clinical usefulness of a shade guide, there are only a limited number of research studies available that have quantified the coverage error of commercially available shade guides in large population samples [18, 19].

The coverage error of dental shade guides can be calculated from visual (${\mathrm{CE}}_{\mathrm{V}}$) or instrumental (${\mathrm{CE}}_{\mathrm{I}}$) color determinations, having both methods some shortcomings. In ${\mathrm{CE}}_{\mathrm{V}}$ determinations, it is difficult to isolate the inherent error of the instrument (color guide $\mathrm{CE}$) from the error attributable to the observer’s subjectivity [20]. On the other hand, when $\mathrm{CE}$ is calculated instrumentally, the shade matching device selects the best shade tab for the target tooth according to its internally stored data set of dental shade guides [20]. The present study has calculated the $\mathrm{CE}$ of two of the most widely used clinical shade guides, combining visual and instrumental color determination methods, in an attempt to determine the contribution of inherent guide error (${\mathrm{CE}}_{\mathrm{I}}$) to the overall visual error.

Currently, the CIEL*a*b* color space and its associated total color differences formulas CIELAB (${\mathrm{\Delta E}}_{\mathrm{ab}}^{*}$) and CIEDE2000 (${\mathrm{\Delta E}}_{00}$) [21] are widely implemented in dental research [22]. Since the coverage error index is defined as the mean of color differences, to provide a clinical perspective on the interpretation of its values, this index can be compared with 50:50% color difference visual thresholds for dentistry, a well-established quality control tools [23] recommended within ISO/TR 28,642:2016 [17].

The aim of this study was to evaluate color agreement, expressed through instrumental and visual coverage error index, between color of in vivo human teeth and dental shade guides. The null hypotheses tested were that (i) instrumental and visual coverage error index of dental shade guides does not exceed 50:50% perceptibility color difference threshold (PT) for dentistry and (ii) instrumental and visual coverage error index of dental shade guides does not exceed 50:50% acceptability color difference threshold (AT) for dentistry.

## Materials and methods

### Participants

A total of 293 volunteers of Spanish Caucasian population were screened for a transversal observational study to determine visually and measure instrumentally the color of their four upper incisors (target teeth). Finally, 195 participants were selected and included in the study according to the following criteria:

- *Inclusion criteria:* adults aged between 18 and 70, whose target teeth were healthy, without restorations and correctly aligned
- *Exclusion criteria:* dental restorations, stains/pigmentations, or any alteration in size and position of target teeth

The age of participants ranged from 18 to 66 (mean age of 29.1 years), with 73.8% of the sample in the range of 18–30 years. The gender distribution was 59.5% female and 40.5% male. The final sample of this study was formed by a total of 735 incisors (380 central incisors and 355 lateral incisors).

All participants received printed information sheets regarding the aims and scope of the study and signed an informed consent form before being included in the study. The experimental protocol followed the guidelines of the Declaration of Helsinki and was approved by the University Ethics Committee (REF: 829/CEIH/2019). In addition, all participants brushed their teeth before the color evaluation, using the same toothbrush model and toothpaste.

### Color evaluation

#### Visual determination

Two dental clinicians, with more than 5 years of experience, performed visual color determinations. Initially, evaluators were screened for color deficiency using the Ishihara test (Kanehara & Co, Ltd, Tokyo, Japan). Both evaluators were qualified with superior color discrimination level according to the test for color discrimination competency in dentistry described within ISO/TR 28,642:2016 [17].

Visual determinations were performed inside a laboratory, with neutral gray walls and in absence of natural light. Visual shade matching was carried out separate and consecutively by each evaluator, being each examiner blinded to the shade color selected by the other. Shade matching was performed using a standardized simulated D65 light source (Smile Lite, No. 6500, Smile Line, Switzerland) and two commercially available shade guides: VITA classical (VC) and VITA 3D-MASTER BG (V3DM) (VITA Zahnfabrik, BadSackingen, Germany). Both evaluators selected from each shade guide the tab whose central area best matched to the color of central area of the middle third of the vestibular surface of each target tooth. The sequence of the shade tab pickup was based on manufacturer’s instructions of each shade guide. In addition, the degree of the shade match between the target tooth and each shade tab was categorized by each evaluator independently according to three levels: poor (P), when the color of the selected shade tab was not considered as an acceptable match to the target tooth; good (G), when the color of the shade tab was not identical to that of the target tooth but the matching was considered acceptable; optimum (O), when the color of the shade tab was considered identical to that of the target tooth. Visual judgements were performed according to the 2-min rule, to avoid the impact of dental dehydration on in vivo dental color [24]. In addition, participants were required to avoid any contact of the tongue with the target teeth, in order to prevent color changes due to dental rehydration [25].

#### Color measurement

Three short-term color measurements were performed individually by each evaluator using the VITA Easyshade® Advance 4.0 spectrophotometer (EAS) (VITA Zahnfabrik, BadSackingen, Germany). The instrument was calibrated before each measurement, and sterile infection control shields were used. Spectrophotometric measurements were made by placing the tip of the device in contact and perpendicularly to the middle third of the vestibular surface of each target tooth. For each measurement, the EAS reported its best match for VC and V3DM guides as well as the chromatic coordinates CIE L*a*b* of the measured target tooth.

The color of the shade tabs from both shade guides was instrumentally measured using the EAS under the same experimental conditions as described for the target teeth.

### Coverage error index

Color differences between each target tooth and each shade tab from both shade guides were computed using the CIELAB $({\mathrm{\Delta E}}_{\mathrm{ab}}^{*})$ and CIEDE2000 (${\mathrm{\Delta E}}_{00})$ [21] total color difference formulas:

$$\begin{array}{c}{\Delta \mathrm E}_{ab}^{*}\text{=}\sqrt{{\left({\mathrm L}_{1}^{*}\text{- }{\mathrm L}_{2}^{*}\right)}^{2}\text{+}{\left({\mathrm a}_{1}^{*}\text{- }{\mathrm a}_{2}^{*}\right)}^{2}\text{+}{\left({\mathrm b}_{1}^{*}-{ \mathrm b}_{2}^{*}\right)}^{2}}\\ {\Delta {\text{E}}}_{00}={\left[{\left(\frac{\Delta {\mathrm L}^{^{\prime}}}{{\mathrm k}_{\mathrm L}{\mathrm S}_{\mathrm L}}\right)}^{2}+{\left(\frac{\Delta {\mathrm C}^{^{\prime}}}{{\mathrm k}_{\mathrm C}{\mathrm S}_{\mathrm C}}\right)}^{2}+{\left(\frac{\Delta {H}^{^{\prime}}}{{\mathrm k}_{\mathrm H}{\mathrm S}_{\mathrm H}}\right)}^{2}+{R}_{T}\left(\frac{\Delta {C}^{^{\prime}}}{{\mathrm k}_{\mathrm C}{\mathrm S}_{\mathrm C}}\right)\left(\frac{\Delta {H}^{^{\prime}}}{{\mathrm k}_{\mathrm H}{\mathrm S}_{\mathrm H}}\right)\right]}^\frac{1}{2}\end{array}$$

**Fig. 1:**
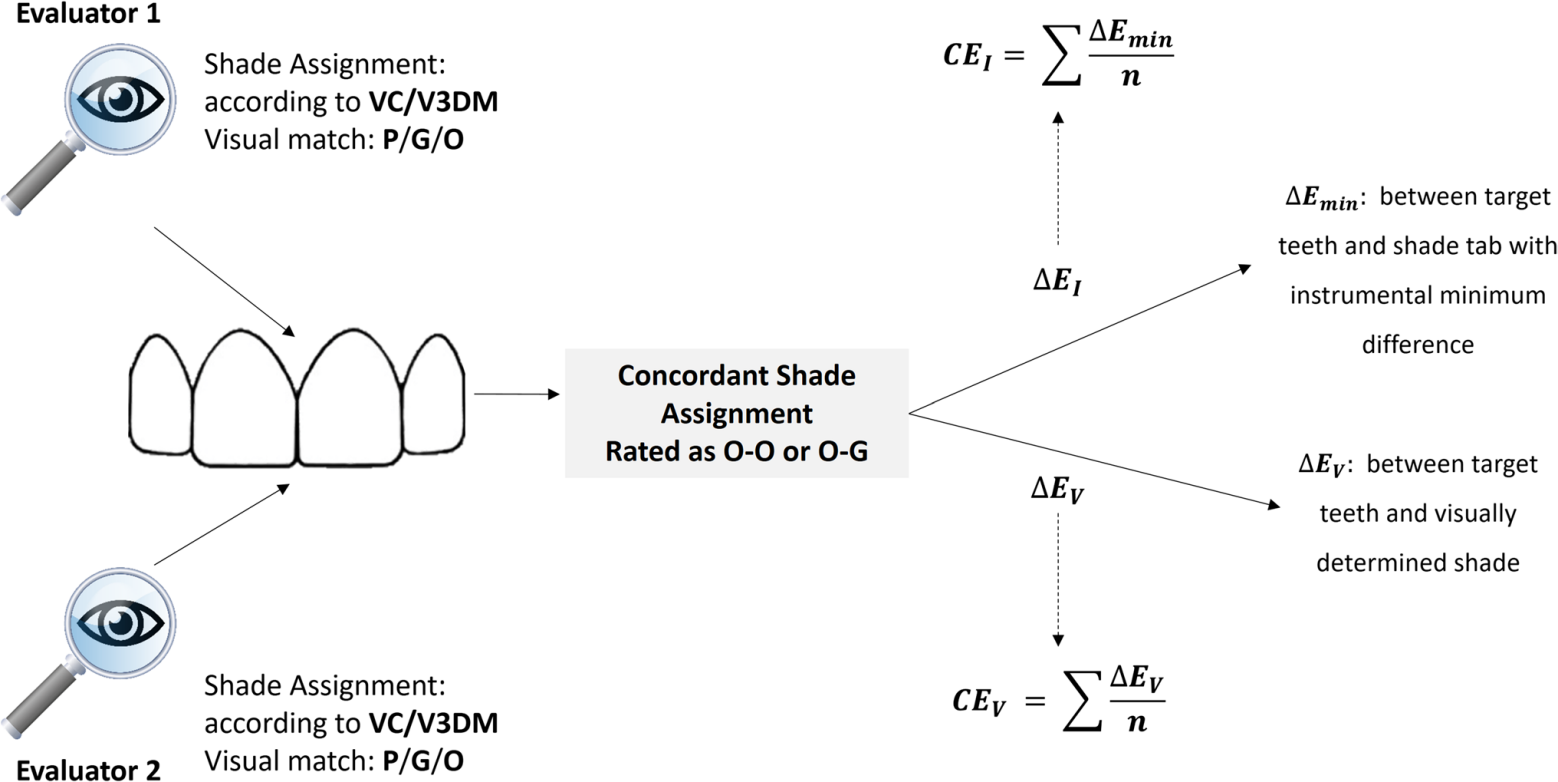
Flowchart for ${\mathrm{CE}}_{\mathrm{V}}$ and ${\mathrm{CE}}_{\mathrm{I}}$ computing

The visual coverage error index (${\mathrm{CE}}_{\mathrm{V}}$) was calculated using $\mathrm{CE}$ index formula [17] as the average of the $\Delta \mathrm{E}$ between each target tooth and its corresponding best matching shade tab as selected by the evaluators:

$$\mathrm C\mathrm E=\sum \frac{{\Delta \mathrm E}_{\mathrm min}}{\mathrm n}$$

where $\Delta \mathrm{E}$ was ${\Delta \mathrm{E}}_{\mathrm{ab}}^{*}$ or ${\Delta \mathrm{E}}_{00}$ according to the color difference formula used; ${\mathrm{CE}}_{V}^{*}$ and ${\mathrm{CE}}_{V}^{^{\prime}}$ then correspond to use of ${\Delta \mathrm{E}}_{\mathrm{ab}}^{*}$ and ${\Delta \mathrm{E}}_{00}$, respectively (Fig. 1).

The ${\mathrm{CE}}_{\mathrm{V}}$ was computed only for concordant inter-observer visual determinations that were rated either as optimum match by both evaluators (O–O), or one evaluator rated it as optimum, while the other rated it as a good match (O–G). This criterion naturally infers on the value of *n* used for ${\mathrm{CE}}_{\mathrm{V}}$ determination: pairs classified by the two evaluators as optimum match (O–O) (*n* = 12 for VC and *n* = 25 for V3DM) and pairs classified as optimum match by one evaluator and good match by the other (O–G) (*n* = 83 for VC and *n* = 58 for V3DM).

In addition, the associated instrumental coverage error (${\mathrm{CE}}_{\mathrm{I}}$) was calculated for each shade guide. However, in this case, for ${\mathrm{CE}}_{\mathrm{I}}$ computation, it was considered the average of the minimum $\Delta \mathrm{E}$ obtained after comparing each selected target tooth with all the shade tabs (the best instrumental matching shade tab).

The 50:50% perceptibility (PT) and acceptability (AT) thresholds for color difference in dentistry described on literature (PT, ${\mathrm{\Delta E}}_{\mathrm{ab}}^{*}$= 1.2 (CI, 0.5–1.9), ${\mathrm{\Delta E}}_{00}$= 0.8 (CI, 0.3–1.3); AT, ${\mathrm{\Delta E}}_{\mathrm{ab}}^{*}$= 2.7 (CI, 2.0–3.4), ${\mathrm{\Delta E}}_{00}$= 1.8 (CI, 1.2–2.4)) [26] were used to interpret the results [17].

## Results

**Fig. 2:**
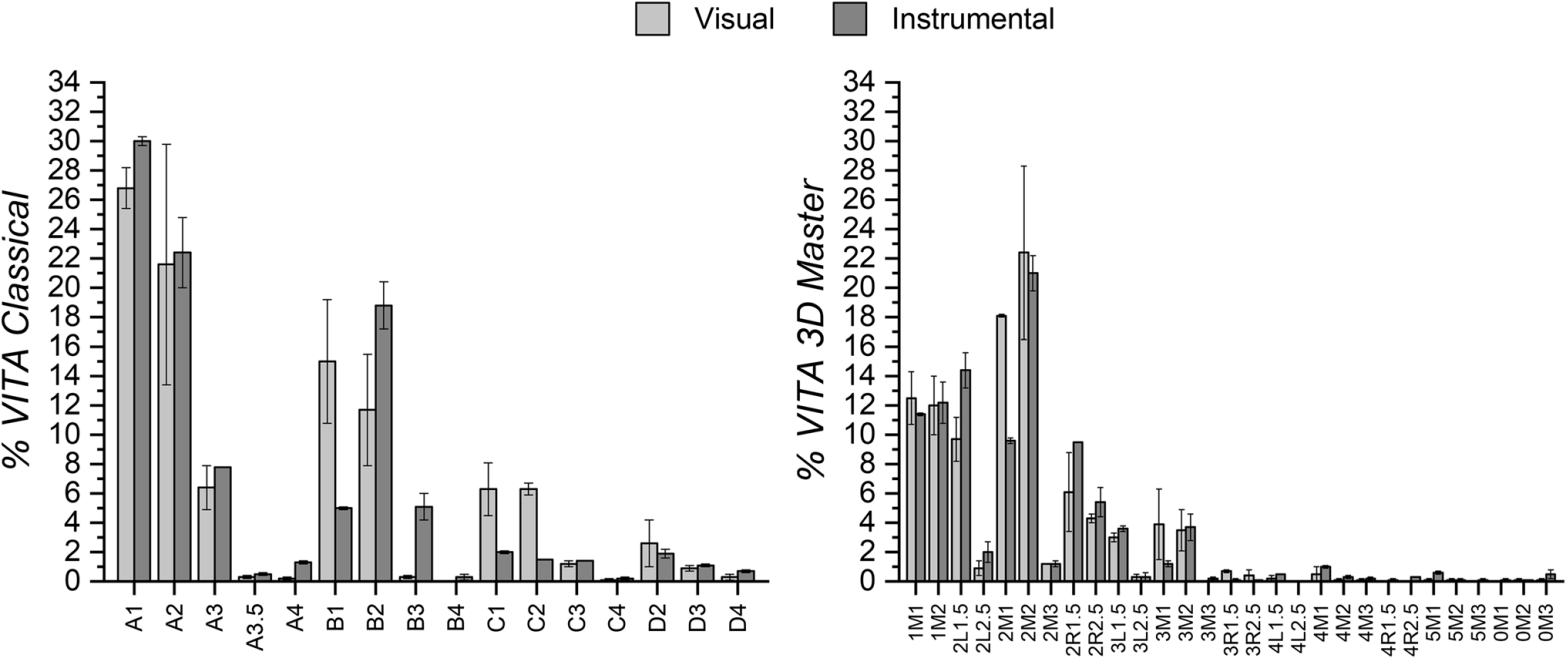
Mean frequency and SD of color shade tab distribution visually determined and instrumentally measured according to VITA Classical guide (left) and VITA 3D Master guide (right)

Figure 2 shows the frequency of shades determined by the evaluators and determined by the EAS, for both VC and V3DM.

For both shade guides, lighter shades were the most selected, with similar trend for visual determinations and EAS measurements. Thus, A1 (26.8%), A2 (21.6%), B1 (15.0%), and B2 (11.7%) were the most frequent shade tabs matched for VC, which correspond to the 75.1% of the total sample. For V3DM, shade tabs 2M2 (22.4%) and 2M1 (18.1%), followed by 1M1 (12.5%) and 1M2 (12.0%) and 2L1.5 (9.7%), were the most selected, which represent the 74.6% of the total sample.

Shade tabs B4 and C4 were the least frequent for VC (0.2%), while 4L2.5 shade tab was not chosen in any case.

Regarding the distribution of the total shade matches according to the match degree (P, G, and O), a similar trend was found for VC and V3DM. The 69.4% and 71.0% of visual determinations were rated as G, 18.1% and 18.9% as O, and P was the least frequent, corresponding to only 12.5% and 10.1% of the matched pairs, respectively.

For each shade guide, the mean frequencies of O–O and O–G pairs of teeth shades with shade agreement between both evaluators were calculated (Table 1). From the total visual determinations performed by the evaluators, only 4.4% for VC and a 10.4% for V3DM were classified as optimum by both evaluators. A 34.6% of the visual determinations (Table 1) were classified either as O–O or O–G, for both guides.

**Table 1:** CE_V_ and CE_I_ mean values and standard deviations (SD) of pairs with shade agreement between both evaluators and O–O and O–G match. The *n* column shows the number of pairs and the percentage (%) of the whole sample they represent

	Visual determinations	$n$	${\mathrm{CE}}_{\mathrm{V}}^{*}$	${\mathrm{CE}}_{\mathrm{V}}^{\mathrm{^{\prime}}}$	${\mathrm{CE}}_{\mathrm{I}}^{*}$	${\mathrm{CE}}_{\mathrm{I}}^{\mathrm{^{\prime}}}$	${~}^{{\mathrm{CE}}_{\mathrm{I}}^{*}}\!\left/ \!{~}_{{\mathrm{CE}}_{\mathrm{V}}^{*}}\right.$	${~}^{{\mathrm{CE}}_{\mathrm{I}}^{\mathrm{^{\prime}}}}\!\left/ \!{~}_{{\mathrm{CE}}_{\mathrm{V}}^{\mathrm{^{\prime}}}}\right.$
VC	O–O	12 (4.4%)	4.7 (2.6)	3.3 (1.7)	2.5 (1.0) *	1.9 (0.7)	53.2%	57.6%
	O–G	83 (30.2%)	4.8 (2.2)	3.4 (1.5)	3.2 (1.7)	2.3 (1.2)	66.7%	67.6%
V3DM	O–O	25 (10.4%)	4.1 (1.8)	3.4 (1.1)	3.2 (1.1)	2.8 (0.7)	78.0%	82.4%
	O–G	58 (24.2%)	4.6 (2.1)	3.6 (1.3)	2.7 (0.9)	2.4 (0.8)	58.7%	66.7%

*Values under AT

${\mathrm{CE}}_{\mathrm{V}}^{*}$, ${\mathrm{CE}}_{\mathrm{I}}^{*}$, ${\mathrm{CE}}_{\mathrm{V}}^{\mathrm{^{\prime}}}$, and ${\mathrm{CE}}_{\mathrm{I}}^{\mathrm{^{\prime}}}$ values of the shade-teeth pairs rated by both evaluators as O–O and/or O–G are included in Table 1. $\mathrm{CE}$ values were similar for both shade guides but slightly higher when using ${\mathrm{\Delta E}}_{\mathrm{ab}}^{*}$. The values obtained for the ${\mathrm{CE}}_{\mathrm{V}}$ for the pairs with O–O rating were similar to the values obtained for those matching pairs rated as O–G, with a maximum difference of 0.5 units registered for V3DM when using ${\mathrm{\Delta E}}_{\mathrm{ab}}^{*}$. In general, all the $\mathrm{CE}$ values obtained were higher for pairs with O–G matches, which highlights that the O–O match was better objectively. The two shade guides included in this study exhibited visual and instrumental coverage error index values greater than 50:50% AT for both color difference formulas used, and only VC exhibited ${\mathrm{CE}}_{\mathrm{I}}^{*}$ smaller than 50:50% AT when using ${\mathrm{\Delta E}}_{\mathrm{ab}}^{*}$ and only when O–O rated matching pairs were considered. For both clinical shade guides and color difference formulas, the ${\mathrm{CE}}_{\mathrm{V}}$ was higher than ${\mathrm{CE}}_{\mathrm{I}}$. The ${\mathrm{CE}}_{\mathrm{I}}/{\mathrm{CE}}_{\mathrm{V}}$ percentages are also shown in Table 1.

## Discussion

It has been shown that the color range and distribution of human teeth are not adequately reflected by nowadays commercially available shade guides [27]. Coverage error index, which represents the mean value for the minimal color differences between specimens of different sets [17], is a useful parameter to compare color ranges and distribution of shade guides and human teeth [12]. The present study explores visual (${\mathrm{CE}}_{\mathrm{V}}$) and instrumental (${\mathrm{CE}}_{\mathrm{I}}$) coverage error of two of the most frequently shade guides used in dentistry (VC and V3DM) on a sample of 735 incisors belonging to 195 volunteers.

A successful dental esthetic restoration involves a total integration of the restorative material with the adjacent natural dental structures. The analysis of color difference findings using visual thresholds greatly supplements the interpretation of research and clinical outcomes. To perceive a color difference and whether that color difference is acceptable is of great importance. Thus, color differences should be evaluated through comparisons with 50:50% perceptibility (PT) and 50:50% acceptability (AT) thresholds [23].

A study [28] evaluated the color of the middle third of 1064 teeth in 133 human subjects and reported that the ${\mathrm{\Delta E}}_{\mathrm{ab}}^{*}$ $\mathrm{CE}$ for VC shade guide was 4.1 units. Other authors reported even higher ${\mathrm{\Delta E}}_{\mathrm{ab}}^{*}$ $\mathrm{CEs}$ for VC (7.2 units) and for V3DM (8.4 units), in an Indian population using digital photography [29]. Both studies utilized different color-measuring devices in different populations, but the results revealed that the $\mathrm{CEs}$ of commercially available shade guide exceeded by far the corresponding acceptability threshold.

A recent study [20] assessed the coverage error and the coverage error percentage for VC and V3DM calculated as the color difference between each tooth region of the participant and matched shade of the shade guide. For VC shade guide, the reported coverage error was 3.0 ± 1.2 ${\mathrm{\Delta E}}_{00}$ units and 3.3 ± 1.3 ${\mathrm{\Delta E}}_{\mathrm{ab}}^{*}$ units, while for the V3DM guide, the coverage error was 2.5 ± 1.3 ${\mathrm{\Delta E}}_{00}$ units and 2.9 ± 1.5 ${\mathrm{\Delta E}}_{\mathrm{ab}}^{*}$ units. The authors concluded that the use of these shade guides may cause inaccurate shade determination due to their high coverage error values.

In our study, visual determinations and instrumental color measurements were made under standardized conditions of illumination and observation/measurement geometry, thus avoiding the possible implications that these factors can have, as pointed out by other authors [30]. In addition, all visual determinations were done using the exact same shade guides, and only one spectrophotometer was used, in order to avoid biases due to manufacturing errors [31]. Evaluators received a specific training before starting the study even though they had broad clinical experience in visual color judgment. In addition, to our knowledge, this study has been the first to categorize the quality of the visual shade match by two independent evaluators according to three different levels (O, G, and P).

The accuracy and reproducibility of the EAS for colorimetric data measurement have been reported to be higher than 90% [10, 32]. However, when performing instrumental measurements on in vivo teeth, one of the disadvantages of this type of clinical spectrophotometers lies in the difficulty of correctly positioning the measuring tip (flat) on the tooth surface (convex) [3].

The EAS software has the ability to provide a best match shade tab (from VC and V3DM) for the measured dental structure, based on reference colorimetric data stored internally on the device. However, this reference colorimetric data may differ from the colorimetric values of the shade guides used for clinical visual determinations. In this sense, all the shade tabs corresponding to both shade guides used in this study were measured, in order to obtain their color coordinates.

The sample of the dental color space analyzed in our study was representative for a young population due to the higher percentage of participants between 18 and 30 years old (73.8%). Similar results to this work were reported in another study [33], where A1 was the most popular shade (followed by A2 shade) in a young population (18–20 years) with a fair skin color. Nevertheless, other study [34] reported that for a young population (25–35 years old), whose etiology is not specified, A3 was the most prevalent shade, followed by B2 and A2.

Regarding V3DM, our results were close to the results obtained in a recent study on a young (16–30 years) Korean population [35]. However, there are available studies that reported 2R1.5 shade as the most representative (followed by 1M2 and 1M1) [34] or 1M1.5 and 1M2 for a Spanish population between 16 and 30 years old [36].

Chromatic distribution for both guides (Fig. 2) corresponds mainly to light and low-saturated teeth, which agrees with results of previous studies [34–36], as most of the teeth (> 75.0%) match into the lightness groups 2 and 1 of V3DM guide, being consistent with the average age (29.1 years) of the participants in the study.

In our study, $\mathrm{CE}$ was calculated using both ${\mathrm{\Delta E}}_{\mathrm{ab}}^{*}$ and ${\mathrm{\Delta E}}_{00}$ total color difference formulas. ${\mathrm{\Delta E}}_{\mathrm{ab}}^{*}$ has been the most used in dentistry for years; however, ${\mathrm{\Delta E}}_{00}$ incorporates specific corrections for the heterogeneity of the CIELAB color space [19, 37] and is currently CIE recommended formula for total color difference computation [21].

According to the results of our study, independently of the color difference formulas used or the dental shade guide evaluated, ${\mathrm{CE}}_{\mathrm{V}}$ and ${\mathrm{CE}}_{\mathrm{I}}$ values were always higher than their corresponding PT and, in most cases, even than the AT [26]. Therefore, the first null hypothesis of this study must be rejected, but the second null hypothesis was partially rejected.

Despite the values of ${\mathrm{CE}}_{\mathrm{I}}$ were in general higher than their respective AT [26], our results (Table 1) are considerably lower than the ones reported in other study [19], where a ${\mathrm{CE}}_{I}^{*}$ for the V3DM guide of 6.2 CIELAB units was obtained for a sample of 933 teeth belonging to an American population with an age range between 21 and 30 years old. Similarly, other study [18] evaluated the $\mathrm{CE}$ of V3DM for a total of 2,067 teeth belonging to a German population, finding a ${\mathrm{CE}}_{I}^{*}$ of 6.2 CIELAB units for the whole sample and a ${\mathrm{CE}}_{I}^{*}$ value of 5.2 CIELAB units for an age range of 21 to 40 years old. Thus, they reported that the $\mathrm{CE}$ increases according to the age, attributing this result to a greater difficulty in determining color in more chromatic (older) teeth. They also performed the calculations of $\mathrm{CE}$ of the V3DM for the whole sample when intermediate shades are also considered, and the ${\mathrm{CE}}_{I}^{*}$ obtained was similar (6.0 CIELAB units), concluding that a shade guide with intermediate values does not improve the ${\mathrm{CE}}_{I}^{*}$, neither the shade matching. The lower values found in our study for ${\mathrm{CE}}_{\mathrm{I}}$ may originate from the fact that, for ${\mathrm{CE}}_{\mathrm{I}}$ calculation, solely concordant shade assignments with high visual ratings (O–O and O–G) were considered. It is likely that a poor visual rating is to be caused by a higher shade tab-target teeth chromatic difference, so including these pairs in the analysis would increase the ${\mathrm{CE}}_{\mathrm{I}}$ value.

Since the human teeth color range is not adequately reflected in current shade guide color distribution [27], any shade guide has an inherent ${\mathrm{CE}}_{\mathrm{I}}$, which has to be considered as the minimum ${\mathrm{CE}}_{\mathrm{V}}$ possible in the best conditions. According to our results, ${\mathrm{CE}}_{\mathrm{I}}$ contributes more than 50% (53.2–82.4% range) to the ${\mathrm{CE}}_{\mathrm{V}}$ value (Table 1). This contribution depends on the shade guide and the color difference formula used for the calculation of $\mathrm{CE}$. Thus, CIEDE2000 showed higher percentages of the contribution than CIELAB color difference formula, and V3DM shade guide showed higher contribution than VC considering together O–O and O–G determination ratings. A higher contribution of the instrumental $\mathrm{CE}$ to the visual $\mathrm{CE}$ translates into a better ability of the user to approach the intrinsic instrumental error of the shade guide (to be as good as possible with the shade guide used). Additionally, the contribution of the ${\mathrm{CE}}_{\mathrm{I}}$ of the shade guides to the ${\mathrm{CE}}_{\mathrm{V}}$ could also justify that the visual coverage error is greater than the visual color threshold (PT and AT) for dentistry [23].

Finally, in terms of limitations, the use of a contact spectrophotometer involves inaccuracies due to the “edge loss” phenomenon. Tooth curvature also generates color measurement inaccuracies when using non-contact devices, which can be minimized by repeated measurements but cannot be completely eliminated [20]. In addition, the results of this study can be extended in future research to other shade guides, populations, and age groups.”

In this study, both visual and instrumental coverage errors of two of the most widely used dental color shade guides were calculated. The visual coverage error of each shade guide is derived from its inherent instrumental error and the contribution of the subjective visual error attributed to the observer. In the future, these findings can be applied to the development of improved shade guides, with a visual coverage error at or lower than the acceptability color difference threshold for dentistry. Consequently, along the visual and instrumental determination of color, shade diagram, written comments, and clinical dental photographs should be used during laboratory communication, in order to consistently achieve adequate esthetic results.

## Conclusions

Within the limitations of this study, the shade guides exhibited visual coverage errors above acceptability thresholds. Since a large contribution of the instrumental coverage error of the shade guides to the visual coverage error index was observed, it is necessary to further improve the shade guides to guarantee a successful esthetic result of dental restorations.

## References

[1] Simionato A, Pecho OE, Della Bona A (2020). Efficacy of color discrimination tests used in dentistry. J Esthet Restor Dent.

[2] Witkowski S, Yajima ND, Wolkewitz M, Strub JR (2012). Reliability of shade selection using an intraoral spectrophotometer. Clin Oral Investig.

[3] Gómez-Polo C, Gómez-Polo M, Celemin-Viñuela A, Parga MVD, JA,  (2014). Differences between the human eye and the spectrophotometer in the shade matching of tooth colour. J Dent.

[4] Paravina RD, Aleksić A, Tango RN (2021). Harmonization of color measurements in dentistry. Measurement.

[5] Knezović Zlatarić D, Illeš D, Ž Alajbeg I, Žagar M (2016) In vivo evaluations of inter-observer reliability using VITA Easyshade ® Advance 4.0 Dental Shade-Matching Device. Acta Stomatol Croat 50:34–39. 10.15644/asc50/1/5 10.15644/asc50/1/5 PMC5017278 27688424

[6] Brandt J, Nelson S, Lauer HC (2017). In vivo study for tooth colour determination—visual versus digital. Clin Oral Investig.

[7] Hardan L, Bourgi R, Cuevas-Suárez CE (2022). Novel trends in dental color match using different shade selection methods: a systematic review and meta-analysis. Materials.

[8] Llena C, Lozano E, Amengual J, Forner L (2011). Reliability of two color selection devices in matching and measuring tooth color. J Contemp Dent Pract.

[9] Dozić A, Kleverlaan CJ, El-Zohairy A (2007). Performance of five commercially available tooth color-measuring devices. J Prosthodont.

[10] Kim-Pusateri S, Brewer JD, Davis EL, Wee AG (2009). Reliability and accuracy of four dental shade-matching devices. J Prosthet Dent.

[11] Lehmann KM, Igiel C, Schmidtmann I, Scheller H (2010). Four color-measuring devices compared with a spectrophotometric reference system. J Dent.

[12] O’brien WJ, Boenke KM, Groh CL (1991). Coverage errors of two shade guides. Int J Prosthodont.

[13] Rubiño M, García JA, Jiménez del Barco BL, Romero J (1994). Colour measurement of human teeth and evaluation of a colour guide. Color Res Appl.

[14] Hasegawa A, Ikeda I, Kawaguchi S (2000). Color and translucency of in vivo natural central incisors. J Prosthet Dent.

[15] Chu SJ, Trushkowsky RD, Paravina RD (2010). Dental color matching instruments and systems. Review of clinical and research aspects. J Dent.

[16] Samra APB, Moro MG, Mazur RF (2017). Performance of dental students in shade matching: impact of training. J Esthet Restor Dent.

[17] International Organization for Standardization (2016) ISO/TR 28642 (2016). Technical report (E): dentistry - guidance on color measurements.

[18] Haddad HJ, Salameh Z, Sadig W (2011). Allocation of color space for different age groups using three-dimensional shade guide systems. Eur J Esthet Dent.

[19] Yuan JCC, Brewer JD, Monaco EA, Davis EL (2007). Defining a natural tooth color space based on a 3-dimensional shade system. J Prosthet Dent.

[20] Tabatabaian F, Khezri AS, Ourang SAH, Namdari M (2021). Assessment of coverage error for two common commercial dental shade guides using a spectrophotometric method. Color Res Appl.

[21] The International Commission on Illumination (2019). CIE 015:2018 Colorimetry.

[22] Pérez MM, Pecho OE, Ghinea R (2019). Recent advances in color and whiteness evaluations in dentistry. Curr Dent.

[23] Paravina RD, Pérez MM, Ghinea R (2019). Acceptability and perceptibility thresholds in dentistry: a comprehensive review of clinical and research applications. J Esthet Restor Dent.

[24] Ruiz-López J, Pulgar R, Lucena C (2021). Impact of short-term dental dehydration on in-vivo dental color and whiteness. J Dent.

[25] Hatırlı H, Karaarslan EŞ, Yaşa B (2021). Clinical effects of dehydration on tooth color: how much and how long?. J Esthet Restor Dent.

[26] Paravina RD, Ghinea R, Herrera LJ (2015). Color difference thresholds in dentistry. J Esthet Restor Dent.

[27] Paravina RD (2009). Performance assessment of dental shade guides. J Dent.

[28] Paravina RD, Majkic G, Imai FH, Powers JM (2007). Optimization of tooth color and shade guide design: clinical research. J Prosthodont.

[29] Rao D, Joshi S (2018). Evaluation of natural tooth color space of the Indian population and its comparison to manufacturer’s shade systems. Contemp Clin Dent.

[30] Clary JA, Ontiveros JC, Cron SG, Paravina RD (2016). Influence of light source, polarization, education, and training on shade matching quality. J Prosthet Dent.

[31] King KA, Derijk WG (2007). Variations of L*a*b* values among Vitapan® classical shade guides. J Prosthodont.

[32] Znanstveni Rad I, Zlatarić D, Illeš D, Ž Alajbeg I, Žagar M (2015) In vivo and in vitro evaluations of repeatability and accuracy of VITA Easyshade ® Advance 4.0 dental shade-matching device. Acta Stomatol Croat 49:112–8. 10.15644/asc49/2/4 10.15644/asc49/2/4 PMC4988825 27688393

[33] Vadavadagi SV, Kumari KV, Choudhury GK (2016). Prevalence of tooth shade and its correlation with skin colour - a cross-sectional study. J Clin Diagn Res.

[34] Rodrigues S, Shetty SR, Prithviraj DR (2012). An evaluation of shade differences between natural anterior teeth in different age groups and gender using commercially available shade guides. J Indian Prosthodont Soc.

[35] Kim H-K, Hee-Kyung Kim C (2018). A study on the color distribution of natural teeth by age and gender in the Korean population with an intraoral spectrophotometer. J Esthet Restor Dent.

[36] Gómez-Polo C, Gómez-Polo M, de Parga JAMV, Viñuela AC (2015). Study of the most frequent natural tooth colors in the Spanish population using spectrophotometry. J Adv Prosthodont.

[37] Pecho OE, Ghinea R, Alessandretti R (2016). Visual and instrumental shade matching using CIELAB and CIEDE2000 color difference formulas. Dent Mater.

